# Design of the “EAST” strategy in patients with symptomatic intracranial atherosclerotic stenosis

**DOI:** 10.3389/fneur.2025.1520356

**Published:** 2025-05-09

**Authors:** Jiaqi Dai, Haobo Zhao, Xinyu Chen, Ping Nie, Jie Gong, Min Wang, Kezhong Zhang, Zhaolu Wang, Hua Lu

**Affiliations:** ^1^Department of Neurology, The First Affiliated Hospital of Nanjing Medical University, Nanjing, China; ^2^Department of Radiology, The First Affiliated Hospital of Nanjing Medical University, Nanjing, China; ^3^Department of Neurosurgery, The First Affiliated Hospital of Nanjing Medical University, Nanjing, China

**Keywords:** statin, intracranial atherosclerotic stenosis, treatment, HR-vwMRI, PCSK9 inhibitor

## Abstract

**Introduction:**

There is a high risk of stroke occurrence and recurrence in patients with intracranial atherosclerotic stenosis (ICAS) despite aggressive medical therapy. Evolocumab is a monoclonal antibody which can inhibit proprotein convertase subtilisin-kexin type 9 (PCSK9) and effectively reduce the level of low-density lipoprotein cholesterol. We hypothesize that evolocumab added to statin therapy (EAST) can stabilize intracranial plaques in patients with symptomatic ICAS.

**Methods and analysis:**

This is a prospective, randomized, open-label, blinded end-point study, which will assess the efficacy and safety of evolocumab in patients with symptomatic ICAS. Eighty patients who suffer a stroke/transient ischemic attack (TIA) caused by ICAS recently will be randomly allocated in a 1:1 ratio to the evolocumab plus statin treatment group or the statin treatment group. High resolution vessel wall magnetic resonance imaging (HR-vwMRI) will be performed at recruitment and after 6 months and 12 months. The primary outcome is changes in plaque characteristics assessed by HR-vwMRI at 6th month and 12th month after treatment. Cognitive and neurological function will also be evaluated at recruitment and follow-up. This trial is being conducted at the first affiliated hospital of Nanjing medical university, China.

**Ethics and dissemination:**

All participants will sign written informed consents. Peer-reviewed articles will be published to disseminate study outcomes.

**Clinical trial registration:**

ClinicalTrials.gov, identifier: NCT05741086.

## 1 Introduction

Intracranial atherosclerosis stenosis (ICAS) is one of the important causes of ischemic stroke occurrence and recurrence worldwide and is especially common in Asian (1, 2). It accounts for 30–50% of strokes in Asian, but only 5–10% in the West (3, 4). Aggressive medical management (e.g., high intensity statin therapy to reduce low-density lipoprotein (LDL) cholesterol <1.8 mmol/L) is superior to the percutaneous transluminal angioplasty and stenting for symptomatic ICAS in the SAMMPRIS study. However, around one in five patients still experienced stroke recurrence or death during 3-year follow-up in the aggressive medical treatment group (5).

As a fully human monoclonal antibody and a member of proprotein convertase subtilisin-kexin type 9 (PCSK9) inhibitors, evolocumab has the potential to decrease LDL cholesterol levels by around 60%. The FOURIER study showed that evolocumab decreased cardiovascular events (i.e., cardiovascular death, myocardial infarction, and stroke), in patients with atherosclerotic cardiovascular disease during 2.2 years follow-up (6). It’s still largely unknown whether evolocumab could affect the evolution of ICAS and reduce ischemic events in patients with stroke.

We thus designed this prospective, randomized, open-label, blinded end-point study to evaluate the effects of evolocumab added to statin therapy (EAST) in patients with stroke/transient ischemic attack (TIA) caused by ICAS. Eighty patients will be enrolled and assigned into two arms with or without evolocumab. Changes in plaque characteristics on high resolution vessel wall magnetic resonance imaging (HR-vwMRI), neurological function and cognitive function will be evaluated and analyzed between groups over a period of 1 year follow-up.

## 2 Materials and methods

### 2.1 Study design

**Figure 1:**
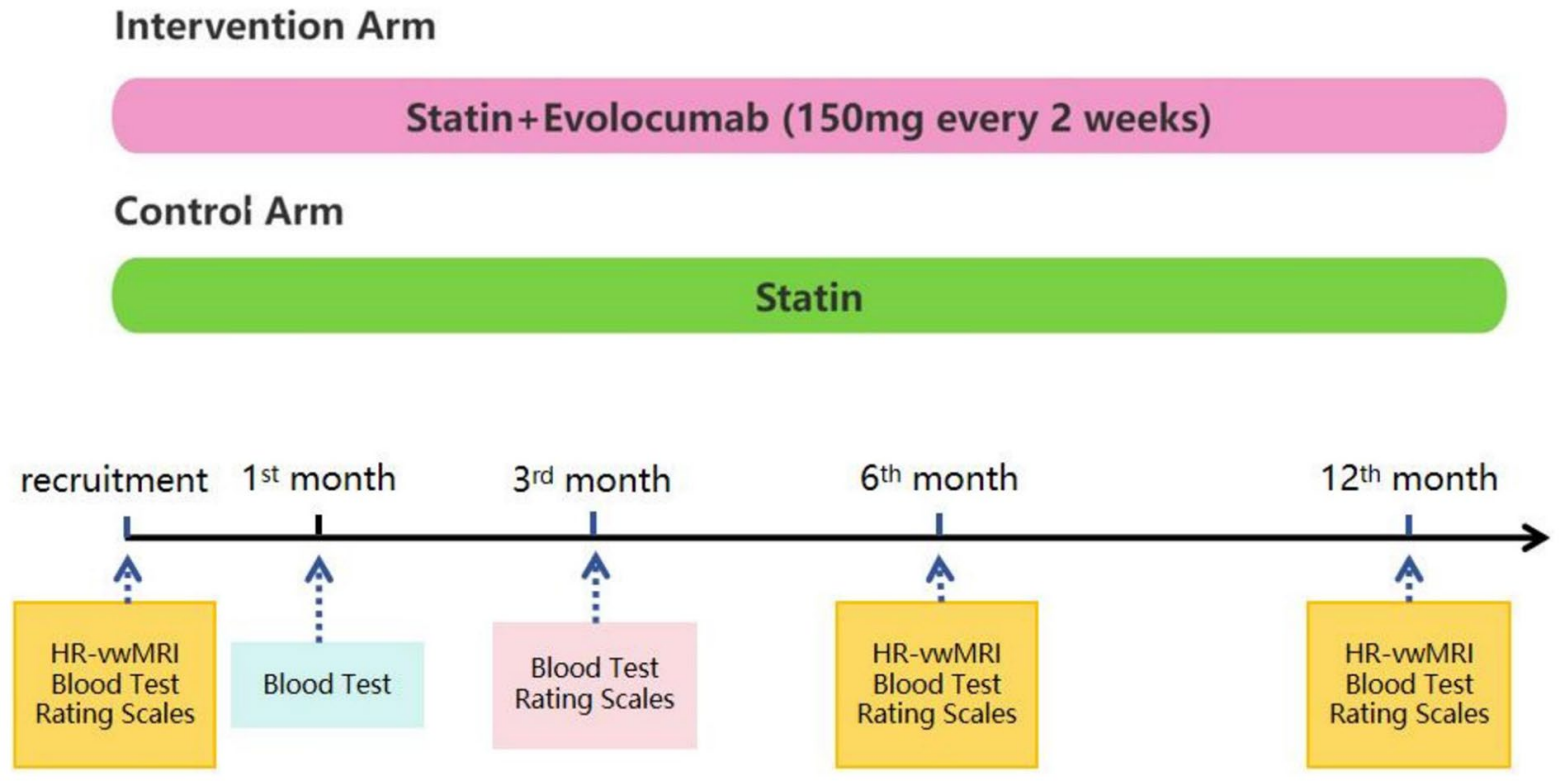
Schematic of the study design and treatment schedule. Statin, including rosuvastatin 10–20 mg or atorvastatin 20–80 mg, will be administered in both arms for 12 months. All patients will receive optimal medical management with aggressive control of the risk factors (e.g., appropriate use of antiplatelet, antihypertensive, and antidiabetic drugs). Patients in the intervention arm will receive an additional treatment of evolocumab 150 mg subcutaneously every 2 weeks for 12 months. All patients will undergo high resolution vessel wall magnetic resonance imaging (HR-vwMRI) at recruitment and after 6 and 12 months. Blood tests will be performed at baseline and at 1, 3, 6, and 12 months after recruitment. Rating scales, including NIHSS, Barthel index, modified Rankin Scale, MoCA, and MMSE, will be used to assess neurological functions and cognitive functions at baseline and at the 3rd, 6th, and 12th months after recruitment.

This is a prospective, randomized, open-label, and blinded endpoint assessment study. We will recruit 80 patients who have recently suffered a stroke/transient ischemic attack (TIA) due to ICAS. The inclusion and exclusion criteria are shown in Tables 1, 2, respectively. Patients will be allocated in a 1:1 ratio to the evolocumab plus statin treatment arm or the statin alone treatment arm. Evolocumab (150 mg) will be injected subcutaneously every 2 weeks for 12 months. Both arms will receive statins for 12 months, including atorvastatin 20–80 mg or rosuvastatin 10–20 mg. According to current guideline and the standards in SAMMPRIS study, optimal medical management with aggressive control of the risk factors will be provided to all patients, such as proper application of antiplatelet, antihypertensive, and antidiabetic drugs. HR-vwMRI will be performed at baseline and at 6th and 12th month after recruitment. Blood test will be conducted at baseline and at 1st, 3rd, 6th, and 12th month after recruitment. National Institute of Health Stroke Scale (NIHSS), modified Rankin Scale (mRS), Barthel index, Mini-Mental State Examination (MMSE), and Montreal Cognitive Assessment (MoCA) will be evaluated at baseline and at 3rd, 6th, and 12th month post-recruitment. This study was approved by the ethics board of the first affiliated hospital of Nanjing Medical University. Written informed consents will be obtained. The flow chart of this study is shown in Figure 1.

**Table 1:** Inclusion criteria.

1. Age ≥30 years and ≤75 years
2. TIA or acute ischemic stroke that occurred within 6 weeks prior to randomization
3. Modified Rankin score of ≤4
4. TIA or acute ischemic stroke attributed to a 50 to 99% stenosis of a major intracranial artery [internal carotid artery (ICA), vertebral artery (VA), basilar artery (BA) and the M1 segment of middle cerebral artery (MCA)]. The diagnostic evaluation for ICAS at each site is confirmed by the local investigator, using high resolution MR
5. To increase the likelihood that the symptomatic intracranial stenosis is atherosclerotic, patients aged 30–49 years are required to meet at least one additional criteria (i–vi) below:
o Insulin dependent diabetes for at least 15 years
o At least 2 of the following atherosclerotic risk factors: hypertension [blood pressure (BP) ≥140/90 or on antihypertensive therapy]; dyslipidemia [LDL ≥130 mg/dL or high density lipoprotein (HDL) <40 mg/dL or fasting triglycerides ≥150 mg/dL or on lipid lowering therapy]; smoking; non-insulin dependent diabetes or insulin dependent diabetes of less than 15 years duration; family history of any of the following: myocardial infarction, coronary artery bypass, coronary angioplasty or stenting, stroke, carotid endarterectomy or stenting, peripheral vascular surgery in parent or sibling who was <55 years of age for men or <65 for women at the time of the event
o History of any of the following: myocardial infarction, coronary artery bypass, coronary angioplasty or stenting, carotid endarterectomy or stenting, or peripheral vascular surgery for atherosclerotic disease
o Any stenosis of an extracranial carotid or vertebral artery, another intracranial artery, subclavian artery, coronary artery, iliac or femoral artery, other lower or upper extremity artery, mesenteric artery, or renal artery that was documented by non-invasive vascular imaging or catheter angiography and is considered atherosclerotic
o Aortic arch atheroma documented by non-invasive vascular imaging or catheter angiography
o Any aortic aneurysm documented by non-invasive vascular imaging or catheter angiography that is considered atherosclerotic
6. Patient agrees with follow-up visits and is available by phone
7. Patient understands the purpose and requirements of the study, can make him/herself understood, and has signed informed consent

**Table 2:** Exclusion criteria.

1. Previous treatment of target intracranial lesion with a stent, angioplasty, or other mechanical devices (e.g., mechanical thrombectomy, coil embolization)
2. Plan to perform angioplasty, stenting, coiling, thrombectomy, endarterectomy or aneurysmal coil embolization for target vessels/plaques. In case that patients who receive surgeries during follow-up, they will still be followed up for 1 year
3. Intracranial tumor (except meningioma) or any intracranial vascular malformation
4. History of any intracranial hemorrhage (parenchymal, subarachnoid, subdural, epidural)
5. Intracranial arterial stenosis due to arterial dissection; moyamoya disease; any known vasculitic disease; viral vasculopathy; neurosyphilis; any other intracranial infection; any intracranial stenosis associated with cerebral spinal fluid pleocytosis; radiation induced vasculopathy; fibromuscular dysplasia; sickle cell disease; neurofibromatosis; benign angiopathy of central nervous system; postpartum angiopathy; suspected vasospastic process; reversible cerebral vasoconstriction syndrome (RCVS); suspected recanalized embolus
6. Presence of any of the following unequivocal cardiac sources of embolism: chronic or paroxysmal atrial fibrillation, mitral stenosis, mechanical valve, endocarditis, intracardiac clot or vegetation, myocardial infarction within 3 months, dilated cardiomyopathy, left atrial spontaneous echo contrast, ejection fraction less than 30%
7. Use of cholesteryl ester transfer protein (CETP) inhibition treatment, mipomersen, or lomitapide within 12 months prior to randomization. Fenofibrate therapy must be stable for at least 6 weeks prior to final screening at a dose that is appropriate for the duration of the study in the judgment of the investigator. Other fibrate therapy (and derivatives) are prohibited
8. Prior use of PCSK9 inhibition treatment before this recruitment
9. Known allergy or contraindication to aspirin, clopidogrel, evolocumab or statin
10. Active peptic ulcer disease, major systemic hemorrhage within 30 days, active bleeding diathesis, platelets <100,000, hematocrit <30, international normalized ratio (INR) >1.5, clotting factor abnormality that increases the risk of bleeding, current alcohol or substance abuse, uncontrolled severe hypertension (systolic pressure >180 mm Hg or diastolic pressure >115 mm Hg), severe liver impairment [aspartate transaminase (AST) or alanine transaminase (ALT) >3× normal, cirrhosis], creatine kinase >5 times the upper limit of normal (ULN) at final screening, severe renal dysfunction, defined as an estimated glomerular filtration rate (eGFR) <20 mL/min/1.73 square meter at final screening
11. Major surgery (including open femoral, aortic, cardiac or carotid surgery) within previous 30 days or planned in the 1 year after enrollment
12. Dementia or psychiatric problem that prevents the patient from relevant evaluation or follow-up reliably
13. Co-morbid conditions that may limit survival to less than 1 year
14. Currently breastfeeding, pregnancy, planning to become pregnant and unwilling to use contraception for the duration of this study
15. Enrollment in another study that would conflict with the current study

### 2.2 Clinical data

We will collect demographic information, vascular risk factors, and blood test results. Vascular risk factors include smoking, hypertension, diabetes, hyperlipidemia, coronary artery disease and previous ischemic stroke. Blood test data include low-density lipoprotein (LDL), high-density lipoprotein (HDL), total cholesterol, triglyceride, urea nitrogen, serum creatinine, aspartate transaminase (AST) or alanine transaminase (ALT), C-reactive protein (CRP), erythrocyte sedimentation rate (ESR), interleukin 6 (IL6), procalcitonin, glycosylated hemoglobin, D-dimmer and fibrinogen (FIB), and blood routine examination.

### 2.3 HR-vwMRI protocol

All images were performed using a 3.0 T magnetic resonance (MR) (Magnetom Verio or Skyra, Siemens, Erlangen, Germany) equipped with a 32-channel or a 20-channel head-matrix coil. The detailed protocol has been described before (7). Briefly, this HR-vwMRI includes sequences as below: three-dimensional time-of-flight MR angiography (3D TOF-MRA), axial DWI (diffusion weighted imaging), 3D T1-weighted SPACE (sampling perfection with application optimized contrast using different angle evolutions), two-dimensional black blood (2D-BB) T2WI, dynamic susceptibility contrast-perfusion weighted imaging (DSC-PWI) conducted with a gradient echo planar sequence using of a bolus of 0.1 mmol/kg contrast agent (gadodiamide, GE Healthcare, Cork, Ireland). In addition, contrast-enhanced 3D T1-weighted SPACE will be acquired with an around 5-min delay time after contrast administration. It takes approximately 25 min to complete all sequences above.

### 2.4 Image analysis

Two neuroradiologists will independently analyze HR-MRI images on commercially available software (Carestream Vue PACS v12.1, Carestream Health), and they were blinded to all clinical information. A senior neuroradiologist will give a further review on images with uncertainty grading to reach a consensus.

The characteristics of the plaque, such as stenosis degree, enhancement, the presence of T1 hyperintensity, remodeling index, plaque burden, and the distribution of the plaque, will be assessed. The detailed imaging analysis on HR-vwMRI has been described before (7). Briefly: (1) degree of stenosis = (1 − D_plaque_/D_reference_) × 100%, where D_plaque_ means the diameter of the stenotic artery at the most stenotic site, and D_reference_ is the diameter of the normal artery proximal to the plaque; (2) grading of plaque enhancement: grade 0 means enhancement is similar to or less than that of normal-appearing intracranial arterial walls in the same subject; grade 1, enhancement is greater than that of grade 0 but less than that of the pituitary infundibulum; and grade 2, enhancement is similar to or greater than that of the infundibulum (8); (3) plaque enhancement ratio (ER): neuroradiologists will draw circular region of interest (ROI) within the plaque on pre-contrast and post-contrast T1-weighted SPACE images, respectively. The mean signal intensity (SI) of plaques will be acquired. ER = (SI_post_ − SI_pre_)/SI_pre_ × 100% (9); (4) remodeling index (RI): the outer wall area (OWA) is manually contoured at the most stenotic site (OWA_plaque_) and the reference site (OWA_reference_) on T1-weighted SPACE. RI = OWA_plaque_/OWA_reference_ × 100%. Arterial remodeling is classified as positive if RI >1.05, intermediate if 0.95 ≤ RI ≤ 1.05, and negative if RI <0.95 (10); (5) plaque burden (PB): lumen area (LA) is manually contoured at the most stenotic site (LA_plaque_) on T1-weighted SPACE. PB = (1 − LA_plaque_/OWA_plauqe_) × 100% (11, 12); (6) presence of T1 hyperintensity: the brightest spot of the plaque whose SI is >150% of that of the reference vessel wall on pre-contrast T1 image (13); (7) plaque distribution: a concentric plaque is defined if the wall involvement is at least 75%, and the minimum wall thickness is bigger than 50% of the maximum wall thickness.

For patients experiencing an acute ischemic stroke within the middle cerebral artery (MCA) territory, infarct patterns on DWI will be categorized into four types: internal borderzone, cortical borderzone, core MCA and perforator (14). For infarction lesion that crossed these territories, the territory which is predominantly involved is chosen. DSC-PWI is derived through the application of the singular value decomposition deconvolution technique, facilitated by the software NeuBrainCARE (v1.1.10). The arterial input function is determined automatically, taken from the MCA on the opposite side. Hypoperfusion volume is automatically calculated using time to maximum (T_max_) with time thresholds of >4 s and >6 s, respectively.

### 2.5 Neurological function and cognitive function

At baseline and then after 3, 6, and 12 months, we will evaluate NIHSS for neurological deficits, Barthel index for activities of daily living, mRS for the degree of disability in daily activities, MMSE, and MoCA for cognitive functions.

## 3 Results

### 3.1 Study outcomes

Primary endpoints:

1) Changes in plaque enhancement.

A reduction of ≥ 1 grading in plaque enhancement after 12 months’ treatment was regarded as relief of plaque enhancement. For example, a patient’s plaque enhancement reduces to grade 1 or even 0 after 12 months’ treatment, compared to grade 2 at the time of enrollment.

2) Changes in degree of stenosis.
3) Changes in plaque enhancement ratio.
4) Changes in plaque burden.
5) Changes in hypoperfusion volume.
6) T1 hyperintensity.

We define three types of T1 hyperintensity changes at 12 month after recruitment: type A, no changes before and after treatment; type B, disappearance of T1 heperintensity; type C, new T1 hyperintensity.

7) Changes in remodeling index (RI) of the plaque.

Secondary endpoints:

1) Recurrent stroke (ischemic or hemorrhagic) or death during follow-up.
2) Changes in LDL-cholesterol levels.
3) Changes in NIHSS/Barthel index/mRS score.
4) Changes in MoCA/MMSE score.

All changes above before and after treatment in each arm will be calculated and compared between arms.

### 3.2 Adverse events

We shall document any adverse events that happen throughout the trial ensuring that patients receive prompt medical attention at our facilities or at local hospitals. Interim analyses are conducted to facilitate decisions on either halting or proceeding with the trial based on the data that has been accumulated.

### 3.3 Statistical analysis

Appropriate statistical methods, such as Fisher’s exact test or Pearson’s chi-squared test, will be employed for the analysis of categorical data. Continuous variables will be compared by an independent samples Student’s *t*-test or Mann–Whitney *U*-test, as appropriate. We will present the cumulative incidence of stroke or mortality over a 12-month maximum follow-up period using Kaplan–Meier analysis, and we will determine the hazard ratios along with their 95% confidence intervals through the application of Cox proportional hazards modeling and the log-rank test to assess the efficacy of the treatment. Statistical significance is defined as a *p* value of < 0.05.

## 4 Discussion

This prospective, randomized, open-label, blinded end-point study seeks to investigate the impact of evolocumab plus statin on ICAS. We hypothesize that evolocumab added to statin therapy (EAST) will demonstrate greater efficacy in stabilizing intracranial plaques, as assessed by HR-vwMRI, when compared to the arm of statin alone without evolocumab. Multiple plaque features (enhancement, plaque burden, degree of stenosis, remodeling index, T1 hyperintensity) and hemodynamic markers (hypoperfusion volume defined by T_max_) will be compared between patients with and without evolocumab. Additionally, we will also document and compare neurological function, cognitive performance, blood parameters levels, and the incidence of recurrent stroke between arms.

Nowadays, the treatment of ICAS is mainly composed of antiplatelet therapy, lipid-lowering strategies, the modification of risk factors and interventional procedures (15). Statins are the preferred treatment option for lowering LDL-C (16). It has been observed that intensive statin therapy can stabilize symptomatic intracranial atherosclerotic plaques, as evidenced by a reduction in enhancement and stenosis on sequential HR-MRI assessments. In addition, greater reductions in LDL levels are associated with a more pronounced decrease in plaque enhancement (17). However, more than a third of these patients demonstrated either no improvement or a worsening of plaque enhancement and stenosis after high-dose statin therapy, suggesting a lack of or minimal responsiveness to statins (17). This is consistent with earlier findings regarding the progression of ICAS in patients treated with statins (3, 18). Meanwhile, there is an association between high-intensity statin therapy and increased side effects, including myalgia and liver dysfunction, which can result in inadequate treatment adherence. Endovascular treatments such as stenting may be a promising treatment method for ICAS. Nevertheless, the CASSISS study found that there was no significant difference between stenting combined with drug therapy and drug therapy alone in preventing stroke or death (19). As a member of PCSK9 inhibitor family, evolocumab significantly reduces LDL cholesterol levels, and associated with greater plaque regression and stabilization by optical coherence tomography measures of plaque composition (20), reducing the risk of cardiovascular events (6). Therefore, it is plausible that evolocumab added to statin therapy (EAST) could also stabilize intracranial plaques in stroke/TIA patients with ICAS.

In this study, symptomatic ICAS patients will be randomized into the arm of evolocumab plus statin or the arm of statin alone. The evolution of these plaques will be monitored through sequential HR-vwMRI scans at 6-months and 12-months after enrollment. Future multicenter studies with a large sample size are needed to further investigate the efficacy of this “EAST” strategy in symptomatic ICAS.

## 5 Strengths and limitations of this study

This is one of the pioneering studies to evaluate the impact of a PCSK9 inhibitor on plaque evolution in patients with symptomatic ICAS, extending beyond the scope of conventional medical therapy. This study, characterized by its prospective, randomized, open-label, and blinded end-point design, facilitates patient recruitment and keeps costs low. Considering the limited sample size and single-center recruitment, the findings of this study should be applied with caution, and there is a need for future large-scale, multi-center studies to validate the results.
